# Analysis of Risk Factors of Level V Lymphatic Metastasis for Papillary Thyroid Carcinoma with pN1b

**DOI:** 10.1155/2021/5562065

**Published:** 2021-08-18

**Authors:** Chuang Li, Zhao-zhong Meng, Jian-wu Qin, Xin-guang Qiu

**Affiliations:** ^1^Department of Thyroid and Neck, The Affiliated Cancer Hospital of Zhengzhou University, Henan Cancer Hospital, Zhengzhou 450003, China; ^2^Department of Thyroid Surgery, The First Affiliated Hospital, Zhengzhou University, Zhengzhou 450052, China

## Abstract

**Objective:**

To explore the risk factors of level V lymphatic metastasis in papillary thyroid carcinoma (PTC) patients with pN1b.

**Methods:**

Patients were selected if they presented with a suspicious level III or IV lymph node metastasis and underwent surgery by hemi or total thyroidectomy with a lymph node dissection (levels III, IV, VI, and VII). For these patients, if frozen section showed a positive level III or IV node, then levels II and V nodes were resected. Univariate analysis was performed using the chi-square test for some factors, including age, sex, tumor location, multifocal lesions, tumor size, local invasion of primary focus, status of cervical lymphatic metastasis, TNM staging, tumor deposits (independent tumor nodules), and the metastasis to more than 5 central lymph nodes. Then, the factors with statistical significance indicated by the above univariate analysis underwent multivariate analysis.

**Results:**

Univariate analysis indicated that the level V lymphatic metastasis was significantly associated with simultaneous metastases to levels II, III, and IV, simultaneous metastases to levels III and IV, and tumor deposits (all *p* < 0.05), but it was not significantly associated with age, sex, tumor location, multifocal lesions, tumor size, local invasion of primary focus, other cervical lymphatic metastasis, TNM staging, and the metastases to more than 5 central lymph nodes (all *p* > 0.05). Multivariate analysis suggested that the simultaneous metastases to levels III and IV and tumor deposits were the risk factors of level V lymphatic metastasis.

**Conclusion:**

The simultaneous metastases to levels III and IV and tumor deposits are independent risk factors of level V lymphatic metastasis. The patients with pN1b PTC who have simultaneous metastases to levels III and IV or/and tumor deposits may have the risk of level V lymph node metastasis.

## 1. Introduction

Papillary thyroid carcinoma (PTC), a common pathological type, accounts for approximately 85% of thyroid cancer [1]. In recent 10 years, PTC incidence has been increasing in the world. In PTC, cervical lymph node metastasis is common with a metastasis rate of as high as 40%–90% [2]. PTC metastasis often occurs in the ipsilateral lymph nodes of the primary lesion and is usually along the following lymphatic course: first, the central region (levels VI-VII), then jugular chain lymph nodes (levels II–IV), and finally the posterior cervical lymph nodes (level V), but skip metastasis may also occur [3]. Jugular chain lymph node metastasis affects local tumor recurrence and disease-free survival [4]. American Thyroid Association guidelines made in 2015 recommended lateral neck lymph node dissection for the patients with biopsy-confirmed jugular chain lymph node metastasis [5]. However, it is still controversial whether the patients with lateral neck lymph node metastasis require further level V lymph node dissection. Preoperative ultrasound and CT usually cannot detect level V lymph node metastasis in the patients with PTC. In this study, we analyzed the risk factors of level V lymphatic metastasis in the PTC patients with pN1b to provide a reference for making treatment decisions.

## 2. Subjects and Methods

All study methods were approved by the Ethics Committee of the Affiliated Cancer Hospital of Zhengzhou University. All the subjects enrolled into the study gave written informed consent to participate.

### 2.1. Subjects

The inclusion criteria were (1) over the age of 18 years, (2) PTC confirmed by preoperative pathology, (3) PTC with pN1b identified by intraoperative frozen pathology, and (4) complete clinical data. The exclusion criteria included (1) other types of thyroid cancer, (2) combined with other malignancies, (3) a history of surgery or radiotherapy on the head and neck, (4) recurrent PTC, and (5) incomplete clinical data.

From January 2013 to December 2016, a total of 649 PTC patients with suspicious lateral neck metastasis underwent hemi- or total thyroidectomy with node dissection (levels III, IV, VI, and VII). If his/her intraoperative frozen pathology displayed pN1b, the patient further received ipsilateral levels II and V lymph node dissection.

### 2.2. Surgery and Main Outcome Measures

Patients underwent color ultrasound examinations for the thyroid and neck before operation. If the color ultrasound showed suspicious metastatic lymph nodes in the levels III or/and IV, such as increased lymph node combined with calcification or liquefaction, all the lymph nodes and fatty tissue in the levels III and IV were removed, and all lymph node dissection specimens in the levels III and IV underwent rapid frozen pathological examination. If the color ultrasound indicated suspicious metastatic lymph nodes on bilateral levels III or/and IV, bilateral levels III and IV lymph node dissections were performed. If intraoperative frozen pathology displayed pN1b, the patient further received ipsilateral levels II and V lymph node dissection. In this study, the lymph node metastasis included micrometastasis (<2 mm) and macrometastasis. All patients enrolled in this study underwent at least radical thyroid resection combined with ipsilateral modified levels II–VII lymph node dissection. The items, including age, sex, tumor location, tumor-multifocal, tumor size, local invasion of primary focus, status of cervical lymphatic metastasis, TNM staging, tumor deposits (independent tumor nodules), and the metastases to more than 5 central lymph nodes were recorded. A lymph node was completely replaced by tumor tissues, which was regarded as tumor deposits. The tumor deposits were identified by H. E pathology of lymph node dissection. Under a microscope, the tumor deposits only contained thyroid malignant tissue without these structures, such as lymph tubes, nerves, or blood vessels.

### 2.3. Statistical Analysis

Statistical analysis was performed using SPSS19.0 software. The chi-square test was used in the comparison of qualitative data. And then, the data with statistical significance indicated by the above chi-square test underwent multivariate analysis using the logistic regression test. Statistical significance was established at *p* < 0.05.

## 3. Results

### 3.1. General Data

pN1b PTC was identified in 132 patients from the 649 PTC patients. Of the 132 patients, 47 were male and 85 female, with a mean age of 42 years (range 19–77). The 132 patients underwent at least radical thyroid resection combined with ipsilateral modified levels II–VII lymph node dissection. In the 132 patients, 50 received ipsilateral modified levels II–VII lymph node dissection, 40 bilateral modified levels II–VII lymph node dissection, and 42 ipsilateral modified levels II–VII lymph node dissection combined with contralateral selective lymph node dissection. The status of cervical lymphatic metastasis is given in Table 1. In the 132 patients, level V lymph node metastasis was found in 14 patients (10.61%, 14/132) (Table 1). The tumor deposits occurred in 19 patients (14.39%, 19/132) (Table 2).

### 3.2. Univariate Analysis of the Level V Lymphatic Metastasis-Related Factors

Univariate analysis indicated that the level V lymphatic metastasis was significantly associated with simultaneous metastases to levels II, III, and IV, simultaneous metastases to levels III and IV, and tumor deposits (all *p* < 0.05), but it was not significantly associated with age, sex, tumor location, tumor-multifocal, tumor size, local invasion of primary focus, other cervical lymphatic metastasis, TNM staging, and the metastases to more than 5 central lymph nodes (all *p* > 0.05) (Table 2).

### 3.3. Multivariate Analysis of the Level V Lymphatic Metastasis-Related Factors

The factors with statistical significance indicated by the above univariate analysis underwent multivariate analysis using the logistic regression test. Multivariate analysis suggested that the simultaneous metastases to levels III and IV and tumor deposits were the independent risk factors of level V lymphatic metastasis (all *p* < 0.05) (Table 3).

## 4. Discussion

The level V lymphatic metastasis is strongly associated with postoperative local tumor recurrence and disease-free survival in PTC [4], but its incidence is relatively low. The level V lymph node dissection may cause accessory nerve and cervical plexus injuries, which may lead to shoulder dysfunction, as well as numbness and neuralgia in the cervical region [6, 7]. At present, little research has been performed on level V lymphatic metastasis in the PTC patients with pN1b. Chen et al. [8] reported that in 106 patients with cN0, 29 received levels II–V lymph node dissection, and level V lymphatic metastasis was found in 2 patients with a metastasis rate of 6.9% (2/29). The 2 patients with level V lymphatic metastasis had simultaneous metastases to levels II, III, and IV, so they recommended level V lymph node dissection for the patients with simultaneous metastases to levels II, III, and IV. Retrospective studies displayed that the level V lymphatic metastasis rate was between 12.3% and 53% in the patients with PTC undergoing II–VI lymph node dissection [9–13]. Terrell et al. [7] found that the level V lymphatic metastasis rate was 16% in the patients who were clinically diagnosed with the negative level V lymph node. Shim et al. [11] reported that the level V lymphatic metastasis rate was 18.2% (26/143) in 143 patients who had lateral neck lymph node metastasis and received surgery for the first time. Wang et al. [12] retrospectively observed 1037 PTC patients with cN1b and found that the level V lymphatic metastasis rate was 21.3% (221/1037). Yang et al. [13] retrospectively analyzed 220 patients with solitary PTC and found that the level V lymphatic metastasis rate was 12.3%. There is also considerable debate about the risk factors of the level V lymphatic metastasis. At present, most researchers believe that multiregion lymphatic metastasis among levels II, III, and IV is associated with the level V lymphatic metastasis, and it is an independent risk factor of the level V lymphatic metastasis [9, 13]. Kupferman et al. [10] reported that the level V lymphatic metastasis was significantly related to tumor-multifocal lesions and ipsilateral lateral neck lymph node metastasis. Shim et al. [11] and Wang et al. [12] found that there was an independent correlation between lymph node-extracapsular spread and the level V lymphatic metastasis. Wang et al. [12] also found that lymph node ≥2 cm, simultaneous metastases to levels II, III, and IV, and unilateral central lymph node metastasis were the independent predictors of the level V lymphatic metastasis (*p* < 0.05). Yang et al. [13] believed that the ipsilateral level V lymphatic metastasis was closely related to the simultaneous metastases to levels II–IV, tumor size >1 cm, extrathyroid invasion, ipsilateral central lymphatic metastasis rate ≥50%, contralateral central lymph node metastasis (CLNM), and bilateral central metastasis, and CLNM was an independent risk factor of the level V lymphatic metastasis.

In the 132 patients, the level V lymphatic metastasis rate was 10.61% (14/132) which was higher than 6.9% reported by Chen et al. [8], similar to 12.3% reported by Yang et al. [13] and slightly lower than the results reported in references [9–16]. The various level V lymphatic metastasis rates may be related to the different indications of level V lymph node dissection. In this study, the PTC patients were diagnosed with positive pN1b by levels III and IV lymph node dissection, so the PTC patients enrolled in this study contained some PTC patients with lateral cervical occult metastasis. Compared with the PTC patients with positive cN+, the course of disease was relatively shorter and the level V lymphatic metastasis rate was lower in this study.

In this study, the level V lymphatic metastasis-related factors underwent univariate analysis and multivariate analysis, and results suggested that the simultaneous metastases to levels III and IV and tumor deposit were the independent risk factors of level V lymphatic metastasis (all *p* < 0.05). PTC metastasis is usually along the following lymphatic course: first, the central region (levels VI and VII), then lateral neck lymph nodes (levels II–IV), and finally the posterior cervical lymph nodes (level V, especially Vb). In this study, the simultaneous metastases to the levels III and IV are an independent risk factor of the level V lymphatic metastasis, which may be that the levels III and IV are closer to the level Vb in space as compared with other lymph nodes. In this study, it was not found that the simultaneous metastases to the levels II, III, and IV was significantly associated with the level V lymphatic metastasis, which may be that the sample size of patients with the simultaneous metastases to the levels II, III, and IV was small and the PTC patients with the level II lymphatic metastasis rarely have level V lymphatic metastasis. This remains to be further confirmed by large-sample clinical studies.

Tumor deposits, a special histopathological feature, were first discovered by Gabriel et al. [17] in rectal cancer in 1935. The tumor deposits are an important risk factor of prognosis in colorectal cancer and gastric cancer. It is defined as an independent presence of tumor nodules without identifiable lymph nodes, lymphatic vessels, nerves, or blood vessels [18]. The tumor deposit formation is not completely clear. It may originate from direct dissemination of tumors, lymph node metastasis, vascular invasion, or nerve invasion, and with tumor progresses, the original lymph node structure is destroyed followed by tumor deposits formation [19]. In AJCC staging, N stage only includes N1a and N1b, without considering the degree of metastatic lymph node invasion [20]. However, the degree of metastatic lymph node invasion may be associated with the risk of PTC recurrence [21]. At present, there are few studies on the tumor deposits of thyroid cancer, but tumor deposit formation is often found in the cervical lymph node dissection specimens. In this study, tumor deposits were found in 19 samples (19/132, 14.39%). The tumor deposits are usually associated with tumor recurrence and distant metastasis. In this study, both univariate analysis and multivariate analysis indicated that tumor deposit formation was significantly correlated with the level V lymphatic metastasis and is an independent risk factor of level V lymphatic metastasis. This suggests that the pN1b PTC patients with tumor deposits easily have lymph node metastasis. The tumor deposits may be regarded as lymph node-extracapsular spread because they have similar biological behaviors [22]. Shim et al. [11] and Wang et al. [12] found that there was an independent correlation between lymph node-extracapsular spread and the level V lymphatic metastasis. If the tumor deposits are regarded as lymph node-extracapsular spread, our results are similar to the results of Shim et al. [11] and Wang et al. [12].

In summary, the patients with pN1b PTC who have simultaneous metastases to levels III and IV, or/and tumor deposit, may have the risk of level V lymph node metastasis.

## Figures and Tables

**Table 1:** Cervical lymph node metastases in different levels in papillary thyroid carcinoma.

Levels	Lymph node metastases (*n*)	Metastasis rate (%)
II	59	44.7 (59/132)
III	123	93.18 (123/132)
IV	93	70.45 (93/132)
V	14	10.61 (14/132)
II and III	53	40.15 (53/132)
II and IV	49	37.12 (49/132)
III and IV	79	59.85 (79/132)
II, III, and IV	44	33.33 (44/132)

**Table 2:** Univariate analysis of the factors associated with the level V lymph node metastases.

Factors	Patients (*n*)	Cervical lymphatic metastasis		*P*
		No	Yes	
Age				
<55	114	101 (88.6%)	13 (21.44%)	0.691
≥55	18	17 (94.44%)	1 (10.44%)	

Sex				
Female	85	76 (89.41%)	9 (19.91%)	1
Male	47	42 (89.36%)	5 (20%)	

Tumor location				
Upper	18	18 (100%)	0 (0%)	0.1
Middle	66	61 (92.42%)	5 (14.24%)	
Lower	6	5 (83.33%)	1 (31.33%)	
Multifocal PTC	34	28 (82.35%)	6 (33.18%)	
Whole lobe	8	6 (75%)	2 (47%)	

Multifocal PTC				
No	83	75 (90.36%)	8 (18.12%)	0.638
Yes	49	43 (87.76%)	6 (23.02%)	

Tumor size				
≤1 cm	34	31 (91.18%)	3 (16.59%)	0.11
1-2 cm	53	50 (94.34%)	3 (10.64%)	
>2 cm	37	32 (86.49%)	5 (25.41%)	
>4 cm	8	5 (62.5%)	3 (70.5%)	

Local invasion				
No	50	45 (90%)	5 (18.8%)	0.105
Thyroid capsule	34	33 (97.06%)	1 (5.53%)	
Outside capsule	48	40 (83.33%)	8 (31.33%)	

Level II, III, and IV metastases				
No	88	83 (94.32%)	5 (10.68%)	0.015^*∗*^
Yes	44	35 (79.55%)	9 (38.45%)	

Level II and III metastases				
No	79	74 (93.67%)	5 (11.9%)	0.051
Yes	53	44 (83.02%)	9 (31.92%)	

Level III and IV metastases				
No	53	53 (100%)	1 (3.55%)	0.007^*∗*^
Yes	79	65 (82.28%)	13 (30.94%)	

Level II and IV metastases				
No	83	75 (90.36%)	8 (18.12%)	0.638
Yes	49	43 (87.76%)	6 (23.02%)	

TNM staging				
I	107	99 (92.52%)	8 (14.06%)	0.12
II	13	9 (69.23%)	4 (57.85%)	
III	8	7 (87.5%)	1 (23.5%)	
IVB	4	3 (75%)	1 (47%)	

Tumor deposits				
No	113	105 (92.92%)	8 (13.31%)	0.006^*∗*^
Yes	19	13 (68.42%)	6 (59.37%)	

Metastases to over 5 central lymph nodes				
No	122	108 (88.52%)	14 (21.57%)	0.599
Yes	10	10 (100%)	0 (0%)	

Level II metastases				
No	73	66 (90.41%)	7 (18.03%)	0.673
Yes	59	52 (88.14%)	7 (22.31%)	

Level III metastases				
No	9	8 (88.89%)	1 (20.89%)	1
Yes	123	110 (89.43%)	13 (19.87%)	

Level IV metastases				
No	39	33 (84.62%)	6 (28.92%)	0.248
Yes	93	85 (91.4%)	8 (16.17%)	

**Table 3:** Multivariate analysis of independent risk factors of level V lymphatic metastasis.

Factors	Regression coefficient	Statistic	Degree of freedom	*P*	OR	95% CI
Tumor deposits						
No	Reference group					
Yes	2.977	11.713	1	0.001^*∗*^	19.627	(3.568–107.955)

Level III and IV metastases						
No	Reference group					
Yes	3.272	6.797	1	0.009^*∗*^	26.374	(2.253–308.712)

Level II, III, and IV metastases						
No	Reference group					
Yes	1.192	2.792	1	0.095	3.295	(0.814–13.344)

Notes: OR, odds ratio; CI, confidence interval. ^*∗*^*P* < 0.05.

## Data Availability

The data used to support the findings of this study are available from the corresponding author upon reasonable request.
